# Supplementary figures and images for: XIAP promotes the expansion and limits the contraction of CD8 T cell response through cell extrinsic and intrinsic mechanisms respectively
Source: PLoS Pathog. 2023 Jun 22;19(6):e1011455. doi: 10.1371/journal.ppat.1011455 (PMC10321636; doi:10.1371/journal.ppat.1011455)

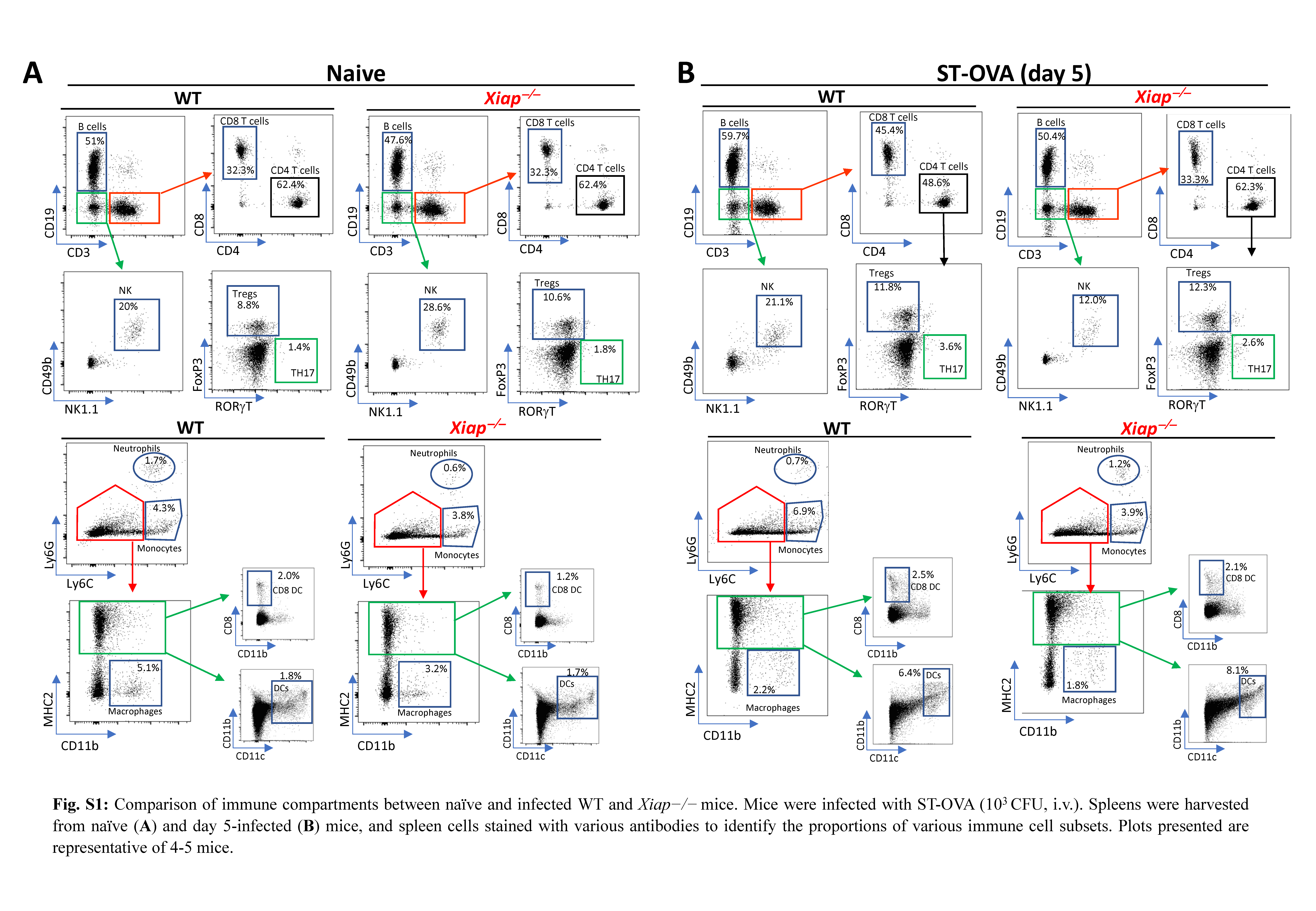

Supplement: S1 Fig — Mice were infected with ST-OVA (103 CFU, i.v.). Spleens were harvested from naïve (A) and day 5-infected (B) mice, and spleen cells stained with various antibodies to identify the proportions of various immune cell subsets. Plots presented are representative of 4-5 mice. (TIFF) [file ppat.1011455.s001.tiff]

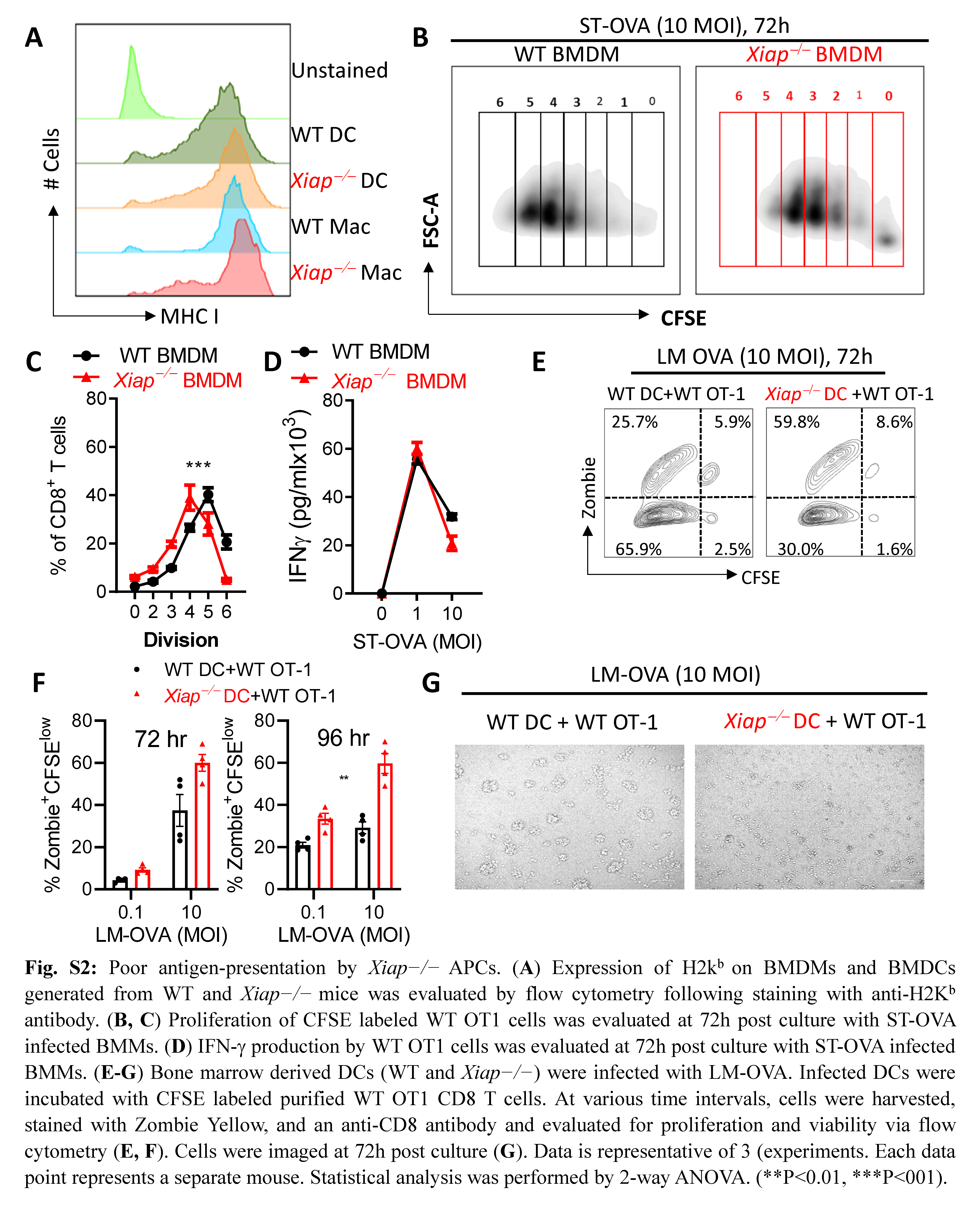

Supplement: S2 Fig — (A) Expression of H2kb on BMDMs and BMDCs generated from WT and Xiap−/− mice was evaluated by flow cytometry following staining with anti-H2Kb antibody. (B, C) Proliferation of CFSE labeled WT OT1 cells was evaluated at 72h post culture with ST-OVA infected BMMs. (D) IFN-γ production by WT OT1 cells was evaluated at 72h post culture with ST-OVA infected BMMs. (E-G) Bone marrow derived DCs (WT and Xiap−/−) were infected with LM-OVA. Infected DCs were incubated with CFSE labeled purified WT OT1 CD8 T cells. At various time intervals, cells were harvested, stained with Zombie Yellow, and an anti-CD8 antibody and evaluated for proliferation and viability via flow cytometry (E, F). Cells were imaged at 72h post culture (G). Data is representative of 3 (experiments. Each data point represents a separate mouse. Statistical analysis was performed by 2-way ANOVA. (**P<0.01, ***P<001). (TIFF) [file ppat.1011455.s002.tiff]

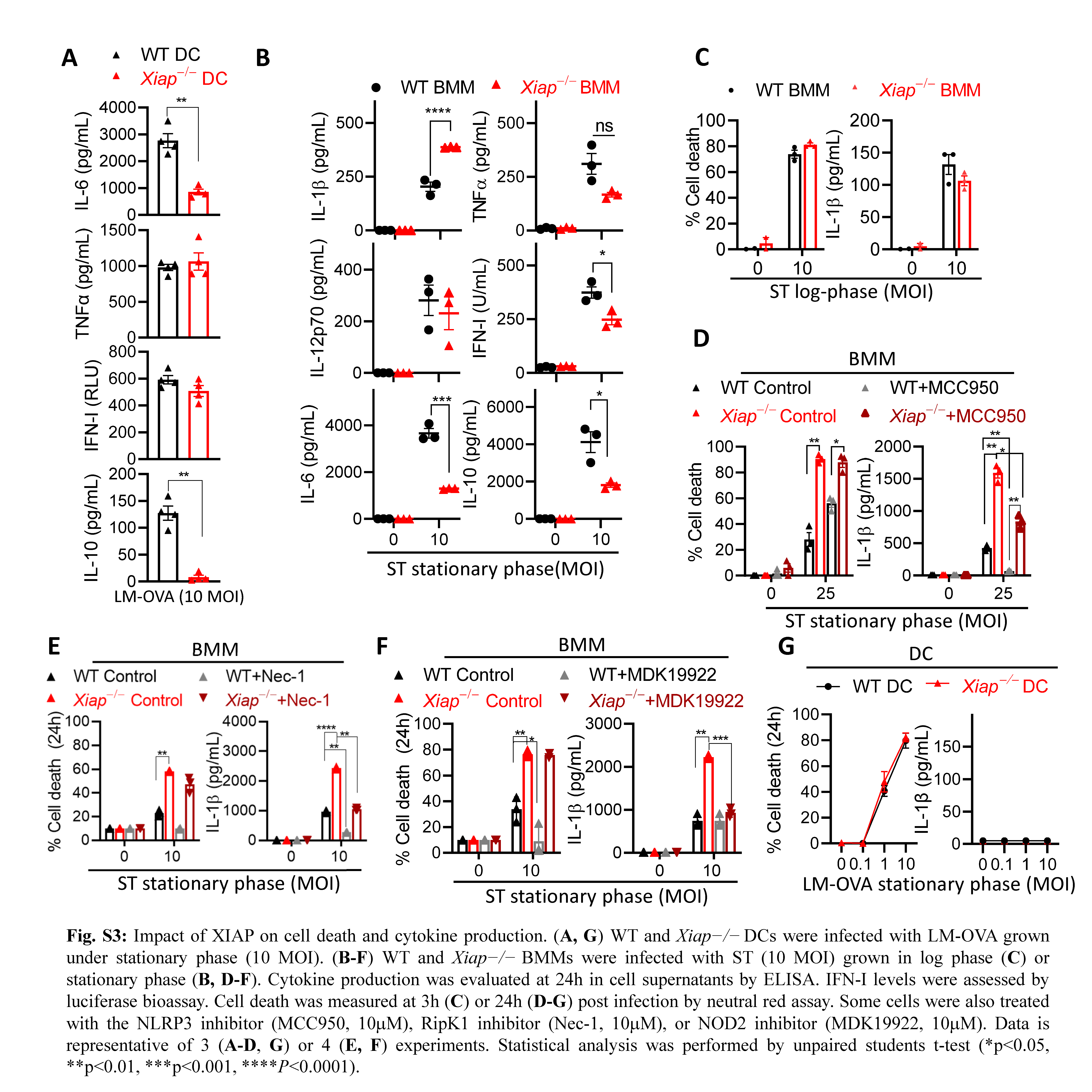

Supplement: S3 Fig — (A, G) WT and Xiap−/− DCs were infected with LM-OVA grown under stationary phase (10 MOI). (B-F) WT and Xiap−/− BMMs were infected with ST (10 MOI) grown in log phase (C) or stationary phase (B, D-F). Cytokine production was evaluated at 24h in cell supernatants by ELISA. IFN-I levels were assessed by luciferase bioassay. Cell death was measured at 3h (C) or 24h (D-G) post infection by neutral red assay. Some cells were also treated with the NLRP3 inhibitor (MCC950, 10μM), RipK1 inhibitor (Nec-1, 10μM), or NOD2 inhibitor (MDK19922, 10μM). Data is representative of 3 (A-D, G) or 4 (E, F) experiments. Statistical analysis was performed by unpaired students t-test (*p<0.05, **p<0.01, ***p<0.001, ****P<0.0001). (TIF) [file ppat.1011455.s003.tif]

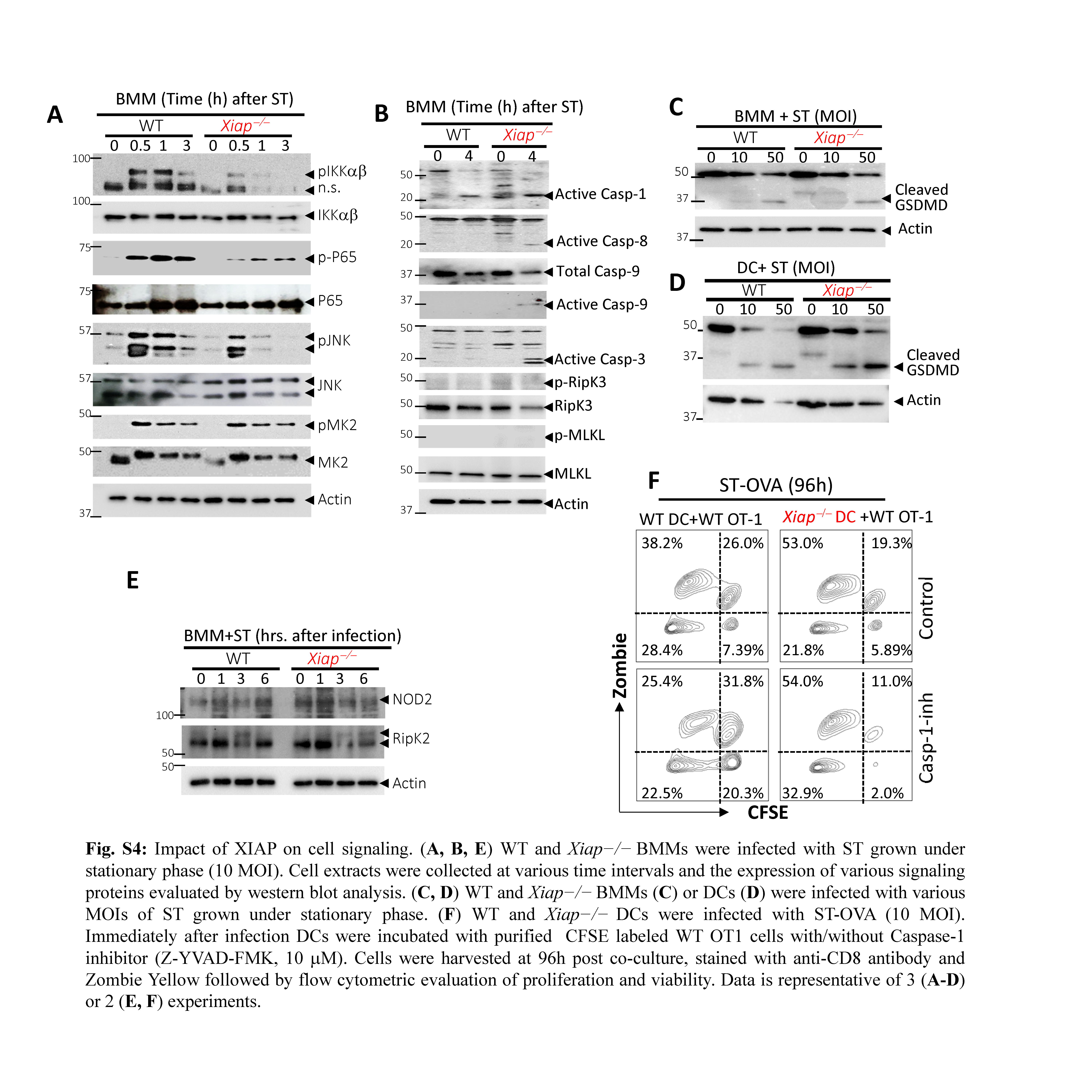

Supplement: S4 Fig — (A, B, E) WT and Xiap−/− BMMs were infected with ST grown under stationary phase (10 MOI). Cell extracts were collected at various time intervals and the expression of various signaling proteins evaluated by western blot analysis. (C, D) WT and Xiap−/− BMMs (C) or DCs (D) were infected with various MOIs of ST grown under stationary phase. (F) WT and Xiap−/− DCs were infected with ST-OVA (10 MOI). Immediately after infection DCs were incubated with purified CFSE labeled WT OT1 cells with/without Caspase-1 inhibitor (Z-YVAD-FMK, 10 μM). Cells were harvested at 96h post co-culture, stained with anti-CD8 antibody and Zombie Yellow followed by flow cytometric evaluation of proliferation and viability. Data is representative of 3 (A-D) or 2 (E, F) experiments. (TIFF) [file ppat.1011455.s004.tiff]

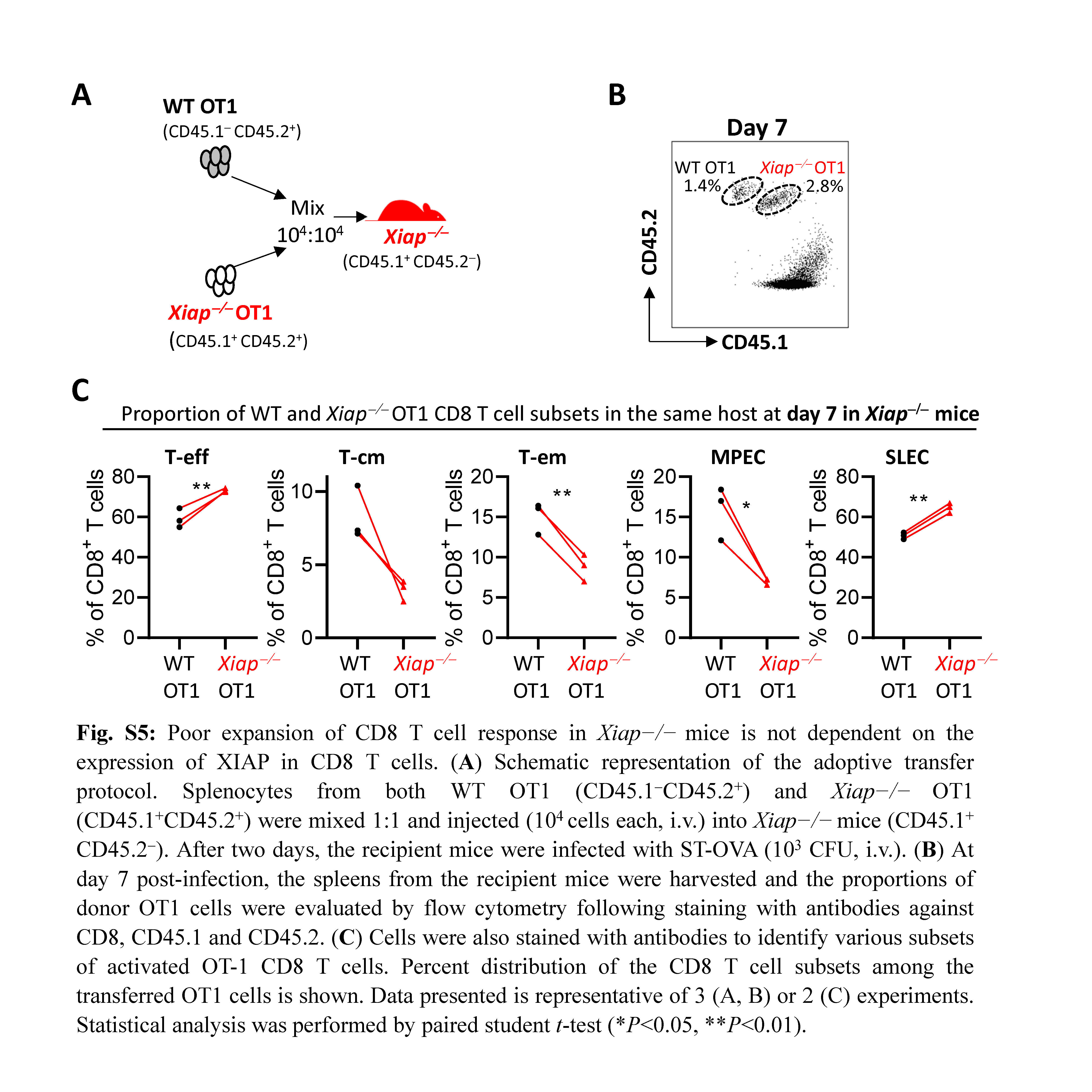

Supplement: S5 Fig — (A) Schematic representation of the adoptive transfer protocol. Splenocytes from both WT OT1 (CD45.1-CD45.2+) and Xiap−/− OT1 (CD45.1+CD45.2+) were mixed 1:1 and injected (104 cells each, i.v.) into Xiap−/− mice (CD45.1+ CD45.2-). After two days, the recipient mice were infected with ST-OVA (103 CFU, i.v.). (B) At day 7 post-infection, the spleens from the recipient mice were harvested and the proportions of donor OT1 cells were evaluated by flow cytometry following staining with antibodies against CD8, CD45.1 and CD45.2. (C) Cells were also stained with antibodies to identify various subsets of activated OT-1 CD8 T cells. Percent distribution of the CD8 T cell subsets among the transferred OT1 cells is shown. Data presented is representative of 3 (A, B) or 2 (C) experiments. Statistical analysis was performed by paired student t-test (*P<0.05, **P<0.01). (TIFF) [file ppat.1011455.s005.tiff]

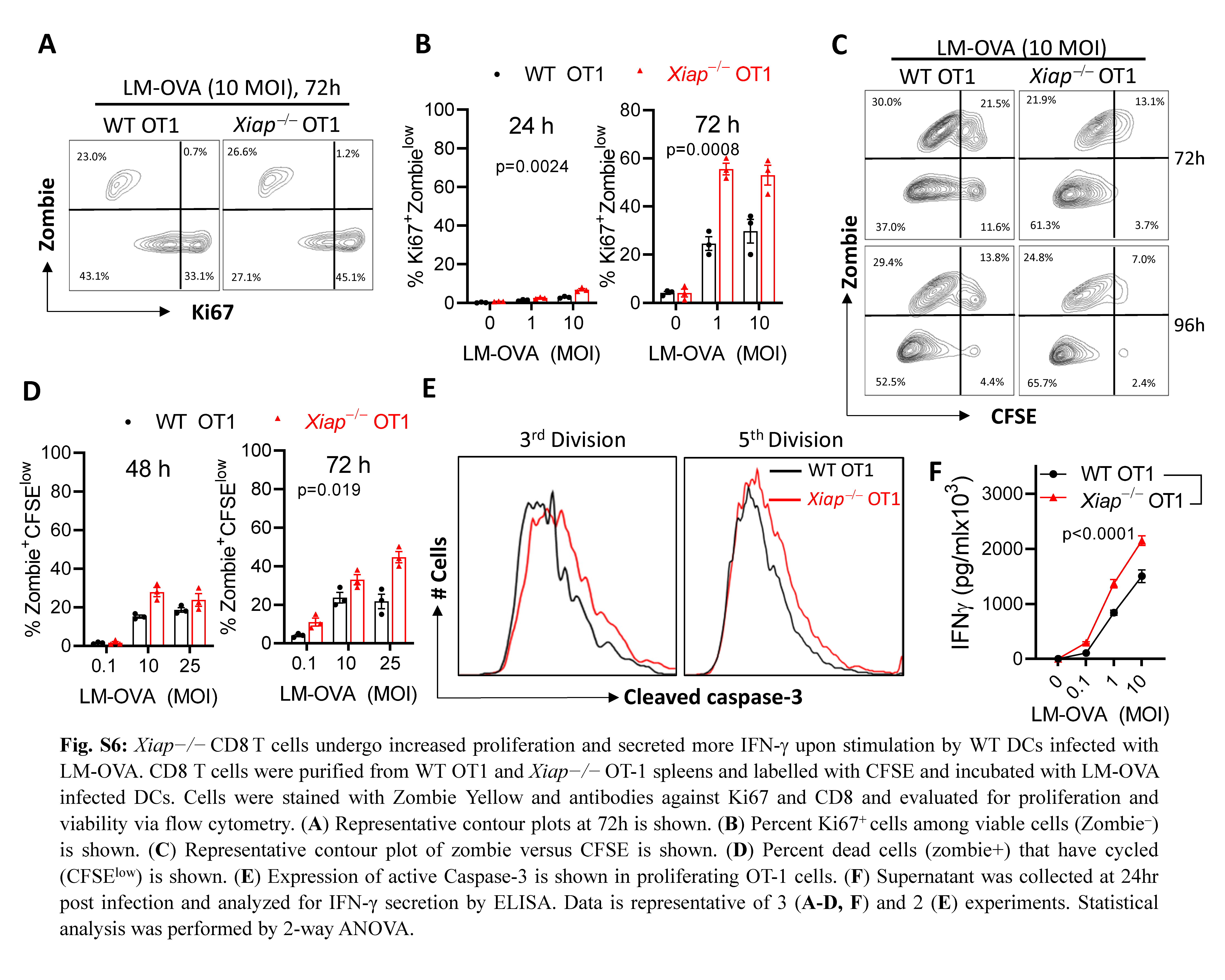

Supplement: S6 Fig — CD8 T cells were purified from WT OT1 and Xiap−/− OT-1 spleens and labelled with CFSE and incubated with LM-OVA infected DCs. Cells were stained with Zombie Yellow and antibodies against Ki67 and CD8 and evaluated for proliferation and viability via flow cytometry. (A) Representative contour plots at 72h is shown. (B) Percent Ki67+ cells among viable cells (Zombie-) is shown. (C) Representative contour plot of zombie versus CFSE is shown. (D) Percent dead cells (zombie+) that have cycled (CFSElow) is shown. (E) Expression of active Caspase-3 is shown in proliferating OT-1 cells. (F) Supernatant was collected at 24hr post infection and analyzed for IFN-γ secretion by ELISA. Data is representative of 3 (A-D, F) and 2 (E) experiments. Statistical analysis was performed by 2-way ANOVA. (TIFF) [file ppat.1011455.s006.tiff]
