# Supplementary material for: XIAP promotes the expansion and limits the contraction of CD8 T cell response through cell extrinsic and intrinsic mechanisms respectively
Source: PLoS Pathog. 2023 Jun 22;19(6):e1011455. doi: 10.1371/journal.ppat.1011455 (PMC10321636; doi:10.1371/journal.ppat.1011455)
Supplement: Attachment — Submitted filename: Point by Point.docx [file ppat.1011455.s008.docx]

**Reviewer's Responses to Questions**

**Part I – Summary**

**Reviewer #1:**

*In their manuscript entitled “XIAP promotes the expansion and limits the contraction of CD8 T cell response through cell extrinsic and intrinsic mechanisms respectively”, Thakker and co-workers specify a novel function of XIAP in the regulation of CD8 T cell reposes towards bacterial infection.*

*Using elaborated T cell mouse models and genetic depletion of XIAP in combination with elegant bacterial infection models, they show that XIAP depletion leads to poor priming and increases expansion and death of CD8 cells resulting in a poor memory response. Moreover, they show that this is partly dependent on extrinsic IL-6 and enhanced cell cycle progression in XIAP ko cells.*

*After its identification as a negative regulator of apoptosis, XIAP was shown to also regulate NF-kB responses, other cell death events and to be an essential component of the NOD1/2 pathway. The integral complexity of the biological function of XIAP that is still not well understood. This is best exemplified by the variance of symptoms associated with disease-associated polymorphisms in XIAP.
In the present work the authors provide a detailed analysis of the function of XIAP in CD8 cell responses towards bacterial infection which is novel and provides a better understanding of role of XIAP in adaptive immunity also in general.
The manuscript is well written and contains a logical flow of very elegant studies to address the role of XIAP in CD8 cells. The reported findings are novel and well substantiated by the data. The methodology applied is very elegant and well suited to address the research question.*
***However, the manuscript might benefit form a somewhat clearer focus. Some parts are rather out of focus and do not provide much novel information, such as the part on antigen presenting cells. Furthermore, issues with data presentation and statistics need to be addressed****.*

***Response:*** *We appreciate the reviewer’s comments about our study. Regarding the role of Xiap on antigen-presentation, we have tried to provide a wholistic picture of the role of Xiap on antigen-presentation and the development of CD8 T cell response. While the impact of Xiap on NFkB and cell death pathways in macrophages has been well established, there isn’t any data that shows that Xiap regulates antigen-presentation through IL-6. Thus, this is a new insight regarding the specific pathways that are regulated by Xiap in different immune compartments.*

*We have removed the data from previous Fig. 3 (panels F-H) on the role of Xiap on antigen presentation in the context of Listeria infection. This dataset has been moved to the Supplemental Fig. 2 (panels E-H). We have also removed previous Fig. 4 which showed the impact of Xiap on cell signalling in macrophages. Parts of this dataset were moved to Fig. 1 (panels A-C), Supplemental Fig. 3 (panels B-D) and Supplemental Fig. 4 (panels A-C).*

*Thus, most of the data that is not novel has been moved to the supplemental section. However, this data is still novel in the context of Salmonella infection.*

*We have also revised the manuscript, added n numbers, performed statistical analysis, and provided experimental details in the legends section of each figure.*

**Reviewer #2:**

*Loss of XIAP function is the genetic cause of immunodeficiency in humans. Multiple murine studies have previously shown that myeloid cells undergo cell death in the absence of XIAP and this could lead to the hyper-inflammatory phenotypes observed in these patients. The role of XIAP in T-cells however has not been as heavily explored. Thus, in this paper, the authors examine the role of XIAP in CD8 T-cell expansion, differentiation and the development of memory. This was done specifically in the context of models for intra-cellular bacterial pathogenic infections by Salmonella enterica (ST) and Listeria monocytogenes (LM).*

*By performing experiments with the ST-OVA, LM-OVA and ST bacterium in both in vivo and in vitro models the authors can show that a general feature of XIAP-deficiency is that CD8 T-cells hyper-proliferate early on in response to infections, and suffer from reduced survival in the long-term. This was not always correlated with bacterial load and depended on the infection model used. As the differences were observed in the early phases of infection the authors conclude that XIAP might play a role in the priming phase.

Based on co-culture experiments with antigen presenting cells (DCs and macrophages), infected with ST-OVA and LM-OVA, WT CD8 OT1 cells proliferated differently. With Xiap-/- APC, the CD8 T-cells underwent fewer cell divisions but more cells underwent division. So the authors conclude, that XIAP-/- myeloid cells promote the rapid expansion of CD8 T cells, that then results in their rapid demise. The authors could pin-point a deficit in IL-6 production from the APCs as a contributing factor to the deregulated CD8 T cell responses, both in vivo and in vitro.*

*In addition to the role of XIAP-deficient APC, the authors could also demonstrate T-cell intrinsic defects of XIAP-deficient CD8 T-cells. These cells showed reduced long-term survival and reduced capacity to generate memory cells. This could be due to defects in IL-2 induction, reduced NF-KB activation and slight increases in multiple caspases. Finally, the authors looked at the long-term impact of this deficit in survival in vitro. Specifically, they look at the capacity of Xiap-/- OT1 cells to generate memory. Looking at consecutive infections of WT mice with ST-OVA and then LM-OVA, the number of Xiap-/- OT1 cells was significantly reduced compared to the WT OT1 cells. In re-infecting mice with ST-OVA the authors could also show that the Xiap-/- mice were unable to control the re-infection as well as mice with WT OT1 CD8 T-cells.*

*In conclusion, the authors suggest that XIAP functions to control CD8 T-cell responses via both extrinsic and intrinsic mechanisms. Extrinsically via the altered cytokine profile of infected antigen presenting Xiap-/- cells, and the intrinsic increase in early proliferation and reduced long-term survival of the CD8 T cells. This results in a deficiency in T-cell memory formation, which could also contribute to the immunodeficiency.*

*These aspects on CD8 biology are novel in the field of XIAP-deficiency and adds to our understanding of the disease mechanism.* ***Much of the data regarding the APCs are not novel and could be pruned down to what is necessary for the T-cell story which is supposed to be the main subject of this manuscript****.*
***However, I find that this paper suffers from poor structure. The result section would benefit from more detailed justifications/explanations behind the experimental setups used. I would also suggest perhaps reducing some of the redundant data for the ST-OVA and LM-OVA models that are just repetitive and do not really add novelty but do make the text more difficult to follow.***

***Response****: The reviewer has summarized our results accurately. As mentioned above for reviewer #1, while the impact of Xiap on NFkB and cell death pathways in macrophages has been well established, there isn’t any data that shows that Xiap regulates antigen-presentation during infection with Salmonella* ***through IL-6*** *which in turn augments CD8 T cell response in a cell extrinsic manner. We have shown this in our experiments in vitro and in vivo.*

*As suggested by the reviewer we have pruned down the impact of Xiap on macrophages. As mentioned in our response to reviewer 1, we have removed previous Fig. 4 which showed the impact of Xiap on cell signalling in macrophages. Parts of this dataset were moved to Fig. 1 (panels A-C), Supplemental Fig. 3 (panels B-D) and Supplemental Fig. 4 (panels A-C). Thus, most of the data that is not novel in terms of what we know about the role of Xiap in antigen presenting cells has been moved to the supplemental section.*

*The redundancy between ST-OVA and LM-OVA has also been addressed. We have removed the data from* ***previous Fig. 3*** *(panels F-H) on the role of Xiap on antigen presentation in the context of Listeria infection. This dataset has been moved to the Supplemental Fig. 2 (panels E-G). While we agree that showing the results in too different experimental models, Salmonella and Listeria, is repetitive, the results provide a comprehensive role of Xiap during infection with intracellular bacteria. Hence the data on antigen-presentation in response to LM-OVA has been moved to the supplemental section.*

*We have tried to improve the structure of the manuscript and have provided justification at the beginning of each result section.*

**Part II – Major Issues: Key Experiments Required for Acceptance**
**Reviewer #1:**

*I would suggest focussing the manuscript on the characterization of the role of XIAP in CD8 cells. The part on antigen-presenting cells does not provide essential information and complicates the story. Also, the abstract would benefit by providing a clearer focus. Moreover, the part on macrophages is rather vague and lacks any detailed analysis of the molecular function of XIAP. The conclusion in line 186 that XIAP promotes IL-6 section in different infection models is not novel and at least analysis of the reported XIAP-dependent pathways, such as NOD1/2 would be wanted.*
***Response:*** *While the impact of Xiap on NFkB and cell death pathways in macrophages has been well established (and we have moved that dataset to the supplemental section), the impact of Xiap on cell signaling during infection with Salmonella has not been reported before. Furthermore, there isn’t any data that shows that Xiap regulates antigen-presentation through IL-6 secretion. The assumption has been that Xiap influences T cell response through a cell-intrinsic mechanism. Thus, the results we have presented provide new insights regarding the specific pathways that are regulated by Xiap in different immune compartments. Furthermore, none of this information has been known in the context of the Salmonella infection model.*

*We have however, removed previous Figure 4 which contained data on the impact of XIAP on various cell signaling pathways in macrophages. Parts of this dataset were moved to Fig. 1 (panels A-C), Supplemental Fig. 3 (panels B-D) and Supplemental Fig. 4 (panels A-C). We have added new data in Fig. S3F which shows that inhibition of NOD2 reduces IL-1β secretion by Xiap^−/−^ but not WT macrophages. In addition, we have added new data in Fig. S3E which shows that inhibition of RipK1-RipK3-interaction reduces IL-1β secretion by both WT versus Xiap^−/−^ macrophages. We have added new data in Fig. S4E regarding the expression of NOD2 and RipK2 in WT versus Xiap^−/−^ macrophages. It has now become clear that pathogens use multiple pathways to induce cell death (PMID: 32735843). Our results showing the impact of various inhibitors supports this view.*

*The manuscript overall is quite descriptive. No details of the molecular mechanisms, for example the role of XIAP in cell cycle, are provided. The authors should at least comment on this a bit more and provide their ideas on possible pathways.*

***Response:*** *It has been previously reported that XIAP ubiquitinates TLE3, the repressor of WNT signaling which results in enhanced WNT signaling. We have commented on this possibility and provided the relevant references on page 18. We have observed that the expression of various WNT targets (Axin2, Lef1, Tcf7) is downregulated in Xiap^−/−^ CD8 T cells (data not shown), but detailed analysis of this will involve substantial more work and will therefore more time will be needed to explore this avenue further.*

***There are critical issues with the data presentation and analysis.*** *All presented figures should include representation of each sample, as provided for same but not all figures. The bar graphs need to be adopted showing all data as points. It is unclear what the authors mean by “Data is representative of 2-3 experiments”. The authors need to indicate for each experiment how often this was independently performed and in how many technical replicates and what of this is shown in the graph. In order to be able to meaningful perform the indicted statistical analysis at least 3-4 independent experiments are needed. In the present form the applied statistical analysis is meaningless for most of the figures. This reviewer suggest that statistical analysis might be omitted if trends are obvious, however the underlying data of each experiment need to be graphed. Experiments that are obviously based in a single data point such in Fig.3 D need to be repeated. This also applies for the supplementary figures.*

***Response:*** *We have revised all the datasets, provided detailed information about the number of experiments in the legends section. We have performed additional experimental repeats and added more replicates in Fig. 1 (d,h,j,k), Fig. 3 (b, d), Fig. 4 (a), Fig. 5 (c), Fig. 6 (c, e, j), Fig. S2 (c, f) and Fig. S6 (b, d). We have also modified the bar graphs to show the data of individual biological repeats.*

**Reviewer #2:** (No Response)

**Part III – Minor Issues: Editorial and Data Presentation Modifications**

**Reviewer #1**:

*- The labelling of the y-axis in Fig.1F is misleading. I would suggest to change to “Ag-specific cells”.*

***Response:*** *This has been changed. Considering the revisions mentioned above, panel F has been changed to panel I.*

*- spaces in front of the citations are missing in lines 287,301 and 377*

***Response:*** *These mistakes have been corrected.*

**Reviewer #2:**

*With virulent ST that kills DCs and Macs in vivo infection of WT and Xiap-/- mice showed a more rapid demise of the Xiap-/- mice. This was associated with reduced CFUs in the Xiap-/- mice and increased IL1b levels and reduced IL6 in the serum. In vitro infection of WT and Xiap-/- BMM with ST also replicated the in vivo cytokine phenotype of higher IL1b and lower Il-6 production from infected Xiap-/- cells. In contrast, infection of BMM with log-phase ST showed no difference in cell death and IL1b production, while stationary phase ST infection again produced this difference in Il1b production. Could this difference in results be due to the fact that log-phase ST directly activates the pyroptotic pathway which has been shown to be intact and normal in Xiap-/- cells, while stationary phase ST is not such a strong canonical activator of pyroptosis?*
***Response:*** *Yes, the explanation provided by the reviewer is in agreement with our interpretation of the data.*

*Figure 2:
It would have been nice to also see the data at day 3/5 post-infection and the pre-infection control to really evaluate whether the reduction in the CD8 T cells is specific to the infection challenge, or is already visible before the infection.*
***Response:*** *We have provided additional data (panel B in Figure 2) which shows similar “take” of OT1 cells in naïve WT and Xiap-/- mice. Given the extent of revisions needed we could not complete the experiments at day 3/5 of infection.*

*Figure 4:
the constant switching between the various stages of ST for infections needs to be made clearer in the text as this impacts the interpretation of the data. For example, it is not clear what phase of ST is used in figures 4G-I.*
***Response:*** *We have now inserted this information in the relevant figure legends. Figure 4 G-I has been moved to Supplemental Figure 4 panels A-C.*

*Figure 5:
In section regarding figure 5, the authors used experiments with either ST or ST-OVA. After showing that the use of stationary or log-phase ST makes a difference in the resulting cytokine expression (Figure 4) this aspect should be made clear in all the other figures using ST and ST-OVA. In figure 5 it is not mentioned at all.*
*Is there an error in Fig. 5A? in the text it is stated that the Xiap-/- DCs die more but in the figure I see that WT DC die more with an MOI of 10.*
***Response:*** *Figure 5 is now Figure 4 because we moved most of the data from previous Figure 4 to the supplemental section. The information on stationary or log-phase ST has been added to figure panels. Thanks for pointing the error in panel B (previously A). We have made the correction.*

*S4A-B:
What is the underlying motivation for looking at TNFR1/2 blockade in this context? Was this experiment done with log/stationary phase ST? and why are such high MOIs now used here? In (B) it looks like a bit more Gasdermin D is cleaved in Xiap-/- DCs, but again why the use of such high MOIs in these experiments?*
***Response:*** *S4A has now been removed and S4B has been moved to S4D now. Since Xiap has been implicated in regulating TNF signaling we considered the possibility that the enhanced death of Xiap^−/−^ macrophages was dependent on TNF-signaling. Cell death was measured at 10 (medium) and 50 (high) MOIs to see there is a rescue at either MOI. We removed this data set based on the comments that a lot of data is redundant and not novel. In our view this data, albeit negative, is interesting. We could include it in the manuscript if the reviewer thinks we should include it. In panel B (now panel D) the Gasdermin appears to be higher in Xiap^−/−^ cells but the actin levels are also higher in Xiap^−/−^ cells at 50 MOI in comparison to WT cells at the same MOI. Hence, it is difficult to interpret.*

*S4C:
Caspase-1 inhibition in WT and Xiap-/- DCs was performed and the impact of this on WT OT1 cell proliferation was examined. The authors state that there was no significant impact of this on CD8 T-cells, but to me it looks like Caspase1 inhibition strongly promoted the proliferation of the T-cells cultured together with WT DCs. Could the authors please comment on that? What impact did caspase-1 inhibition have on the cytokines produced by the DCs and could this explain the effects/lack of effects on the T-cells?*
***Response:*** *This is Figure S4F now. If we look at all the CFSE^low^ cells (+/- Zombie^hi^) in the WT group, the proportion of cells is 66.6% (28.4+38.2) in controls and this number changes to 47.9 (25.4+22.5) upon treatment with the Caspase-1 inhibitor. In the Xiap^−/−^ group the proportion of cells changed from 74.8% to 86.9% following treatment with the Caspase-1 inhibitor. These experiments were not significant. Caspase-1 inhibition resulted in the reduction of IL-1b levels as expected but there was no impact on the expression of other cytokines. This is mainly because of the use of stationary phase bacteria.*

*S4D: why is viability shown for only the MOI 0.1 and 1, but cytokines for up to MOI 10?*
***Response:*** *This is now Fig. S3 G. We have included the cell viability data at 10 MOI as well.*

*Could an explanation for some differences between the ST and LM models be that LM does not primarily target myeloid cells?*
***Response:*** *I think this may be related to the intracellular PAMPS that are engaged by the two pathogens. In case of LM, inflammasome signaling is significantly dependent on AIM2 whereas in case of ST it is dependent on the combination of NLRC4 and NLRP3. These, and other differences may differentially influence the impact of XIAP on the bacterial burden in the two infection models. Salmonella also infects epithelial cells in addition to myeloid cells, but more work needs to be done to dissect why the bacterial burden is highly upregulated in Xiap^−/−^ in the LM model in contrast to the ST model.*

*Line 376/377: XLP2 is an immunodeficiency.*
***Response:*** *We have corrected this sentence (line 381-382)*

*Line 295/296: most studies examining RIPK1 within TNFR1 signaling have found no role for XIAP in ubiquitinylating of RIPK1.*

***Response:*** *We have replaced XIAP with IAPs (line 292).*

**Do you want your identity to be public for this peer review?**

*Reviewer #1: No*

*Reviewer #2: No*
