# Supplementary material for: XIAP promotes the expansion and limits the contraction of CD8 T cell response through cell extrinsic and intrinsic mechanisms respectively
Source: PLoS Pathog. 2023 Jun 22;19(6):e1011455. doi: 10.1371/journal.ppat.1011455 (PMC10321636; doi:10.1371/journal.ppat.1011455)
Supplement: Attachment — Submitted filename: Response to editorial comments.docx [file ppat.1011455.s009.docx]

*Thank you for revising your manuscript according to the issues raised by the Reviewers - both Reviewers are satisfied with these revisions. However, my concern over the status of the mice used in this study was not addressed. Can the authors please indicate whether or not the mice used in the study (eg, Xiap KO vs WT) were littermate animals from a het X het cross?*

***Response:***

**Fig. 1, 2, 3, 4:** Experiments described in these figures were performed with littermates generated from heterozygous breeders.

**Fig. 7 D, E:** Experiments described in these panels were performed with littermates generated from heterozygous breeders.

**Fig. S1-4:** Experiments described in these figures were performed with littermates generated from heterozygous breeders.

**Fig. 5:** In this figure the donor mice were from different cages. WT OT1 (CD45.1−CD45.2+) and *Xiap−/−* OT1 mice (CD45.1+CD45.2+) had to be generated separately based on the differential requirement of CD45.1 versus CD45.2. However, we kept the donor mice in the same cage from the third week of birth (post weaning) to equilibrate the microbiota of the donor mice. These donor cells were mixed (1:1) and transferred to WT mice that were in the same cage.

**Fig. 6:** In this figure the WT OT1 and *Xiap−/−* OT1 mice were from different cages since these mice were generated *for in vivo* tracking experiments. WT OT1 mice were CD45.1−CD45.2+ whereas *Xiap−/−* OT1 mice were CD45.1+CD45.2+. However, we kept these mice in the same cage from the third week of birth (post weaning) to equilibrate the microbiota of mice.

**Fig. 7 A-C:** In these panels the donor mice were from different cages. WT OT1 (CD45.1+CD45.2+) and *Xiap−/−* OT1 mice (CD45.1+CD45.2+) had to be generated separately based on the differential requirement of CD45.1 versus CD45.2. We kept the donor mice in the same cage from the third week of birth (post weaning) to equilibrate the microbiota of the donor mice. These donor cells were mixed (1:1) and transferred to WT mice that were in the same cage.

**Fig. S5:** In this figure the donor mice were from different cages. WT OT1 (CD45.1−CD45.2+) and *Xiap−/−* OT1 mice (CD45.1+CD45.2+) had to be generated separately based on the differential requirement of CD45.1 versus CD45.2. We kept the donor mice in the same cage from the third week of birth (post weaning) to equilibrate the microbiota of the donor mice. These donor cells were mixed (1:1) and transferred to *Xiap−/−* mice that were in the same cage.

**Fig. S6:** In this figure the WT OT1 and *Xiap−/−* OT1 mice were from different cages since these mice were generated *for in vivo* tracking experiments. WT OT1 mice were CD45.1−CD45.2+ whereas *Xiap−/−* OT1 mice were CD45.1+CD45.2+. However, we kept these mice in the same cage from the third week of birth (post weaning) to equilibrate the microbiota of mice.
